# To Our Readers

**Published:** 1891-10

**Authors:** 


### TO OUR READERS.

*The aim of the* JOURNAL *is to furnish matter which will interest its readers, and its policy is to get the best matter available. In pursuance of this policy we introduce a new feature.*

*We desire our readers to write out briefly, and in a practical manner, their method of treating the diseases which shall be mentioned at the head of this page.*

*As the season for* "COMMON COLDS," *or* CATARRHAL FEVER, *will be present for the next few months, we beg you to send in a description of your method of treating this sometimes troublesome ailment. Your letters will appear in order as received.*

*With this, the first issue of the present fiscal year, we send out our regular annual statement to subscribers. We hope these reminders may meet with a ready response at the hands of our patrons.*

